# Induction of the PERK-eIF2α-ATF4 Pathway in M1 Macrophages under Endoplasmic Reticulum Stress

**DOI:** 10.1134/S1607672924600301

**Published:** 2024-07-13

**Authors:** O. E. Kolodeeva, O. E. Kolodeeva, D. A. Averinskaya, Yu. A. Makarova

**Affiliations:** 1 Faculty of Biology and Biotechnology, HSE University, Moscow, Russia; 2 Shemyakin-Ovchinnikov Institute of Bioorganic Chemistry, Russian Academy of Sciences, Moscow, Russia

**Keywords:** viscumin, M1 macrophages, THP-1, ribosome-inactivating protein, PERK-eIF2α-ATF4 pathway, ER stress

## Abstract

Translation inhibition can activate two cell death pathways. The first pathway is activated by translational aberrations, the second by endoplasmic reticulum (ER) stress. In this work, the effect of ribosome-inactivating protein type II (RIP-II) viscumin on M1 macrophages derived from the THP-1 cell line was investigated. The number of modified ribosomes was evaluated by real-time PCR. Transcriptome analysis revealed that viscumin induces the ER stress activated by the PERK sensor.

Viscumin is a plant lectin isolated from *Viscum album*. It is a member of the RIP-II protein family. Another member of this family is the extremely toxic protein ricin [1–3]. RIP-II proteins are heterodimeric glycoproteins consisting of two subunits: A (active) and B (binding), linked by a disulfide bond [4]. The A chain has catalytic activity and hydrolyzes the N-glycosidic bond of adenosine at position 4324 in the sarcin-ricin loop of the 28S rRNA [5–7]. This depurination blocks the binding of the elongation factor EF-2 to the ribosome, resulting in the arrest of protein synthesis in the cell. Irreversible damage of ribosomes activates a ribotoxic stress response (RSR) pathway that ultimately leads to apoptosis or autophagy. B-chain is a lectin that enables binding to specific cellular receptors [8].

The clinical use of viscumin (mistletoe lectin 1) has a century-old history [9, 10], however the mechanism of its therapeutic effect remains unclear. Its antitumor and anti-inflammatory effects have been analyzed [11, 12]. The RIP-II family consists of more than ten proteins. The most studied is ricin. Ricin has been shown to induce ER stress, which plays a significant role in the cell response to the toxin [13]. ER stress occurs in response to the accumulation of unfolded proteins in the lumen and activates the unfolded protein response (UPR) pathway. This pathway alters the transcriptional and translational programs of the cell to cope with stressful conditions [14], in particular triggering the degradation mechanism of misfolded proteins in the proteasome (ERAD, ER-associated degradation) and activating the expression of chaperones in the ER. If viability cannot be restored, the UPR induces apoptosis. There are three main UPR signaling cascades that are initiated by three protein sensors localized in the ER membrane: IRE1a, PERK, and ATF6 [15]. Under physiological conditions, all three sensors are inactive because they are associated with the BiP chaperone (HSPA5/GRP78). This protein binds to unfolded proteins under stress conditions, resulting in their release from the sensor and activation of the sensors. Previously, it has been shown that treatment of cells with ricin activates only the IRE1a-mediated pathway [13].

Proinflammatory M1 macrophages derived from the THP-1 cell line were used as a model to study ER stress, as macrophages play an important role in the immune response and are the major inducers of inflammation. M1 macrophages were treated with viscumin at concentrations ranging from 0.1 to 100 nM. The cytotoxic effect of viscumin was evaluated using the MTT assay. It was found that 50% of cell viability was inhibited (IC50) at a concentration of 4.5 nM viscumin (Fig. 1). A slight increase in cell viability at lower concentrations of viscumin could be due to its mitogenic activity at low concentrations [16]. The sensitivity of macrophages to viscumin was found to be approximately 1000 times greater than that of Caco-2 colorectal adenocarcinoma cells [17].

**Fig. 1.**
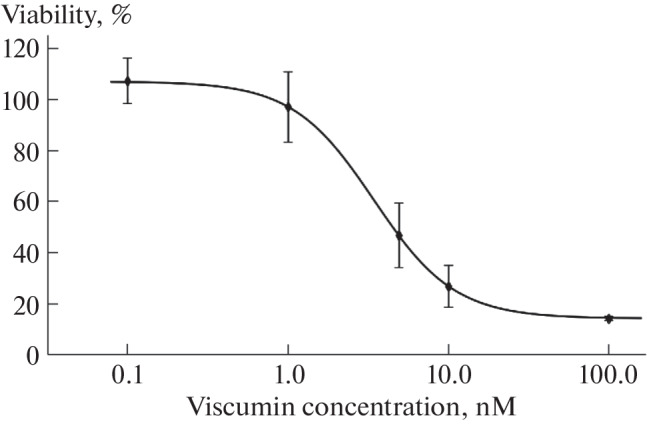
The viability curve of M1 macrophages during treatment of cells with viscumin. The points on the graph represent the average viability value for three biological replicates. Error bars represent the standard error of the mean (SEM). M1 macrophages derived from the THP-1 cell line were treated with viscumin at the indicated concentrations for 6 h, followed by washing and incubation in culture medium for 24 h.

The proportion of ribosomes inactivated by viscumin was evaluated as described previously [17]. In short, the essence of the approach is that the reverse transcriptase inserts deoxyadenine in front of the AP-site formed by the action of the toxin. Therefore, cDNA fragments synthesized on intact and damaged matrices differ by one nucleotide (A instead of T), which can be detected by PCR. A dose-dependent increase in the number of apurinic sites in 28S rRNA was observed (Table 1).

**Table 1.** The proportion of apurinated molecules in the 28S rRNA pool during treatment of M1 macrophages with different concentrations of viscumin for 6 hours and subsequent incubation in a medium without viscumin for 24 hours. The experiment was performed in three biological replicates

Viscumin concentration, nM	The proportion of inactivated 28S rRNA, %
0 (Control)	0 ± 0.00
0.1	0.1 ± 0.01
1	1.2 ± 0.3
10	3.8 ± 0.5
100	6.4 ± 0.6

The proportion of modified ribosomes in M1 macrophages after treatment with viscumin at a concentration of 100 nM, resulting in 85% inhibition of viability, was only 6.45% (Fig. 1, Table 1). Interestingly, as we have previously shown, viability of Caco2 cells does not decrease even after inactivation of 20% of the ribosomes [17]. These data, together with the increased sensitivity of macrophages to viscumin, suggest that the cytotoxic effect of viscumin on M1 macrophages is mediated not only by translation arrest.

Quantitative reverse transcription polymerase chain reaction (RT-qPCR) was used to evaluate the expression levels of ER stress genes: PERK, IRE1a, DDIT3, ATF4, and ATF6 [18, 19]. At high concentrations of viscumin, a statistically significant increase in the expression of all marker genes was observed (Fig. 2).

**Fig. 2.**
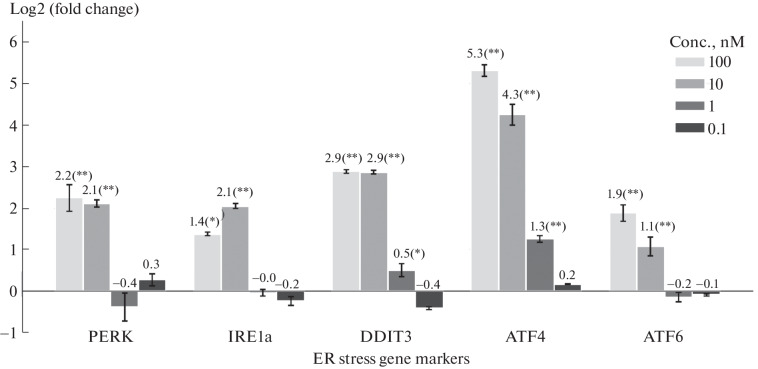
Evaluation of ER stress induction by RT-qPCR. The change of the expression in log2 scale (log2(FC)) in viscumin-treated M1 macrophages at concentrations ranging from 0.1 to 100 nM was evaluated relative to untreated control M1 macrophages using the ∆∆Ct method [20]. Statistical significance of results was assessed by Student’s t-test, adjusted for multiple comparisons by the Benjamini-Hochberg method (* padj < 0.05, ** padj < 0.005). ACTB and GAPDH were used as reference genes. Error bars represent standard error of mean (SEM) of three biological replicates. Polarized M1 macrophages were incubated with the indicated concentrations of viscumin for 6 h, followed by incubation in media without viscumin for 24 h.

To analyze the pathways activated during stress, next generation sequencing was performed on the Illumina platform. Standard bioinformatic analysis included read quality control, adapter trimming, mapping to the human genome, and differential expression analysis. The sequenced samples were treated with viscumin at a concentration of 1 nM (the concentration at which > 50% of the cells remain viable) for 6 h, followed by incubation in medium without viscumin for 24 h. Transcriptome analysis revealed that 742  genes significantly changed their expression (FC  > 1.5, FDR p-value < 0.05): 552 genes were up-regulated and 190 genes were down-regulated. A significant proportion of the overexpressed genes are involved in inflammatory pathways, including the NF-kB pathway.

The expression of genes activated by PERK: ATF4 and DDIT3 (Fig. 3) increased 2.6 and 2.2 times, respectively, which is consistent with the RT-qPCR results (Fig. 2), while the expression of PERK, ATF6, and IRE1a did not change significantly.

For all transcription factors (TF), the changes in the expression of their target genes were analyzed using a hypergeometric test. Table 2 shows the TFs that activate the largest number of target genes. It can be seen that of the three UPR cascades, only the PERK-ATF4 pathway was activated (Table 2).

**Table 2.** TFs that activate the highest number of targets

TF	Number of targets	Odds ratio	FDR
RELA	158	8.22	0.5e-8
NFKB1	150	8.48	0.5e-8
JUN	64	9.84	0.002
HIF1A	43	9.91	0.005
STAT1	36	10.7	0.004
CEBPB	27	19.1	0.0003
ATF4	21	17.1	0.003

**Fig. 3.**
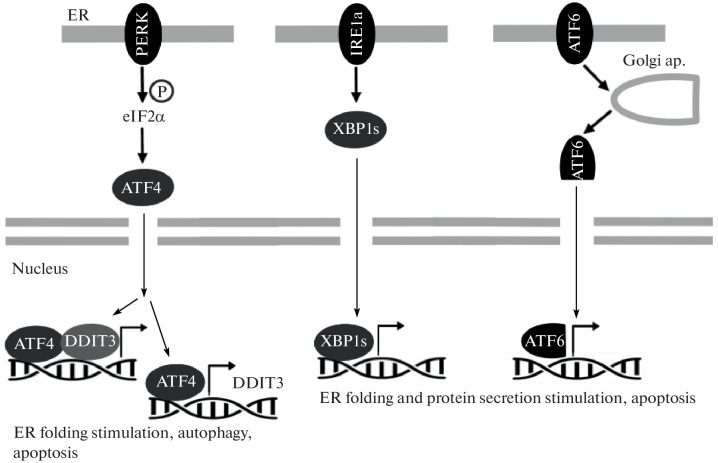
Schematic representation of the UPR signaling pathway that is induced by ER stress. Three UPR cascades activated by PERK, IRE1a, and ATF6 sensors are shown.

ATF4 target genes with increased expression included DDIT3 (FC = 2.1, padj = 4e-11), which is involved in the pathway that activates the PERK sensor, as well as CEBPB (FC = 1.8, padj = 3e-4), ATF3 (FC = 2, padj = 1.6e-39), CXCL8 (FC = 4, padj = 6.7e-26), PPP1P15A/GADD34 (FC = 3.7, padj = 3.2e-125) and SIRT1 (FC = 1.6, padj = 1.9e-20), which activate the inflammatory response and apoptosis. The expression of TFs, which mediate the cellular response during activation of two other ER stress sensors – ATF6 and XBP1 – as well as the expression of their target genes, did not change significantly. This may indicate the considerable contribution of the PERK-eIF2α-ATF4 pathway to the activation of M1 macrophage response mechanisms to viscumin treatment.

Thus, M1 macrophages show a high sensitivity to viscumin, which is probably not only due to the stress caused by ribosomal damage (RSR). Treatment with viscumin leads to activation of the ER stress pathway. Of the three branches of the UPR activated by ER stress sensors, the PERK-EIF2α-ATF4 pathway is the most significant. This may indicate the importance of the PERK sensor in activating the response of M1 macrophages to viscumin exposure. Interestingly, the closely related but more toxic protein ricin has been shown to trigger the pathway activated by the IRE1a sensor, while the PERK-EIF2α-ATF4 pathway is not activated [13]. The activation of different ER stress response pathways by ricin and viscumin may contribute to their different cytotoxicities.

## ABBREVIATIONS AND NOTATION

RIP-II—ribosome-inactivating proteins type II, ER—endoplasmic reticulum, UPR—unfolded protein response, RSR—ribotoxic stress, ERAD—ER-associated degradation, RT-qPCR—quantitative real-time polymerase chain reaction, TF—transcription factor.
